# Design Optimization of Pin Fin Geometry Using Particle Swarm Optimization Algorithm

**DOI:** 10.1371/journal.pone.0066080

**Published:** 2013-05-31

**Authors:** Nawaf Hamadneh, Waqar A. Khan, Saratha Sathasivam, Hong Choon Ong

**Affiliations:** 1 School of Mathematical Sciences, Universiti Sains Malaysia, 11800 USM, Penang, Malaysia; 2 Department of Engineering Sciences, PN Engineering College, National University of Sciences and Technology, Karachi, Pakistan; University of Adelaide, Australia

## Abstract

Particle swarm optimization (PSO) is employed to investigate the overall performance of a pin fin.The following study will examine the effect of governing parameters on overall thermal/fluid performance associated with different fin geometries, including, rectangular plate fins as well as square, circular, and elliptical pin fins. The idea of entropy generation minimization, EGM is employed to combine the effects of thermal resistance and pressure drop within the heat sink. A general dimensionless expression for the entropy generation rate is obtained by considering a control volume around the pin fin including base plate and applying the conservations equations for mass and energy with the entropy balance. Selected fin geometries are examined for the heat transfer, fluid friction, and the minimum entropy generation rate corresponding to different parameters including axis ratio, aspect ratio, and Reynolds number. The results clearly indicate that the preferred fin profile is very dependent on these parameters.

## Introduction

In order to enhance the convective heat transfer from a solid surface, fins of different shapes are used in several applications such as microelectronics, heat exchanger and cooling of engines. The shape of the fin has the main effect on the overall performance and therefore, needs to be optimized for better efficiency. Many researchers including [1]–[9] have studied numerically and experimentally the longitudinal and annular fins of different shapes and determined the overall performance depending upon certain criteria. They found that elliptical fins have a better overall efficiency than other geometries. Khan [10], Poulikakos and Bejan [11], and Culham and Muzychka [12] employed the concept of entropy generation minimization (EGM) and determined theoretically the optimal fin dimensions. EGM combines the fundamental principles of thermodynamics, heat transfer, and fluid mechanics and applies these principles to the modeling and optimization of real systems and processes that are characterized by finite size and finite time constraints, and are limited by heat and mass transfer and fluid flow irreversibilities. They considered different shapes and also obtained the optimal shape for the same parameters to give the better thermal and hydraulic performance. Bar-Cohen and his co-workers [13]–[16] applied a least material optimization technique to plate-fin geometry and extended his analysis to multiple fin arrays. They explored the potential for the least energy optimization of natural and forced convection cooled rectangular plate heat sinks. Chiang and Chang [17] developed an effective procedure to find the optimal values of designing parameters of a pin-fin heat sink. They used the constraints of mass and space limitations and performed several experiments to validate their results.

Genetic algorithms (GAs) have been successfully applied in optimizing heat transfer from solid surfaces. Complex optimization involving non-linear constraints can be easily solved using genetic algorithms. Fabbri [18] proposed a genetic algorithm to optimize the thermal performance of a finned surface. He used finite element method to obtain temperature distribution along the fin and compared the heat flux with that obtained by genetic profile. Copiello and Fabbri [19] optimized heat transfer from wavy fins in forced convection using genetic algorithms. They obtained heat flux by finite element method and optimized fin profile. Hajabdollahi [20] modeled one dimensional heat transfer in a pin fin and optimized it using genetic algorithms. They considered total heat transfer rate and fin efficiency as objective functions and carried out multi-objective optimization to maximize the heat transfer rate and fin efficiency simultaneously. Azarkish et al. [21] obtained the optimal fin geometry for a single fin and fin array. They applied a single objective function GA in a longitudinal fin with 1-D heat transfer. Jha and Chakraborty [22] determined the optimal dimensions of arrays of plate fins in forced convection. They minimized the entropy generation rate using genetic algorithm-based evolutionary computing techniques and investigated the effects of heat transfer and fluid friction on entropy generation rate.

Recently, Particle Swarm Optimization (PSO) technique has attracted several investigators. This technique is one of the most widely used algorithms to find the optimal values in order to minimize the expectation as a function. PSO algorithm and GA operate within a set of solutions, and interactively update it through the application of a number of heuristics [23]. The two algorithms are from different categories, which are trajectory based and non-trajectory based algorithms. PSO is a trajectory based metaheuristic optimization algorithm, which used the concept of distance to update the location of the solutions using a term called velocity. However, GA is not a trajectory based, but rather it switches chromosomes, which are the building block of the solutions, to create a new solution.

PSO algorithm was developed by Kennedy and Eberhart [24] and has been used by Yousefi and Darus [25], Peng et al. [26] and Rao and Patel [27] for the optimization of a cross-flow plate fin heat exchanger. They considered several variables as optimization variables and proved the effectiveness of the proposed algorithm to achieve more accurate results. In another papers, Patel and Rao [28] and Lahiri et al. [29] employed the same technique for the design optimization of shell-and-tube heat exchangers. They considered the minimization of total cost as an objective function. Azarkish et al. [30] compared the performance of both PSO and GA on the geometry of a longitudinal fin. They found that the PSO algorithm is more efficient for geometry optimization. Calçada et al.[23] also showed that the PSO algorithm performed much better than the GA. The Nash equilibrium can be used to understand the PSO algorithm [31], [32]. The Nash equilibrium was conceived to determine optimal strategies in a non-cooperative game. The optimal strategy is a set of strategic choices for the players, such that there is no change in the choice of any single player [33].

The above literature survey reveals that PSO has never been used for optimization of pin fin geometry. The main objective of this study is to optimize pin fins of different shapes using the PSO algorithm, which was proposed by Eberhart and Kennedy [34], [35], with commercial softwer MATLAB® .

### Particle Swarm Optimization Algorithm

The algorithm is initialized with a population of random solutions, and then updated through generating new positions [36]. It is inspired by social behaviour of birds flocking or fish schooling [24], [36], [37]. Furthermore, PSO can be easily implemented; its memory and CPU speed requirements are low. All solution members, so-called particles, flies through the problem space by following the particle with best performance and by tracking their best positions. Each particle will have to position 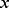, and velocity vectors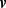, which will be updated with each iteration. PSO algorithm quickly converges to a good solution. However, it is easy to get a local optimal value [38]. When the best particle is known, all other particles are slightly moved during the performance of the best one. In addition, there are also few parameters to adjust. Several fields in engineering and computer science used PSO algorithm [39]–[41].

In PSO algorithm, each particle keeps the best position that it has achieved so far. The particles update their velocities, from the previous velocity using Eq.1. On the other hand, a particle’s position is adjusted according to the Eq.2. 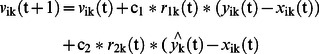
(1)

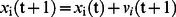
(2)


where


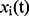
: The position of the particle at time t.


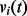
: The velocity of the particle at time t.


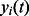
: The personal best position (_p_best) of the particle at time t.


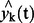
: The global best position (_g_best) for the population.


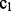
: learning factor of _p_best in interval [0, 2].


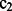
: learning factor of _g_best in interval [0, 2].


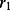
 and 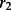 are the random numbers uniformly distributed in interval [0, 1].

**Figure 1:**
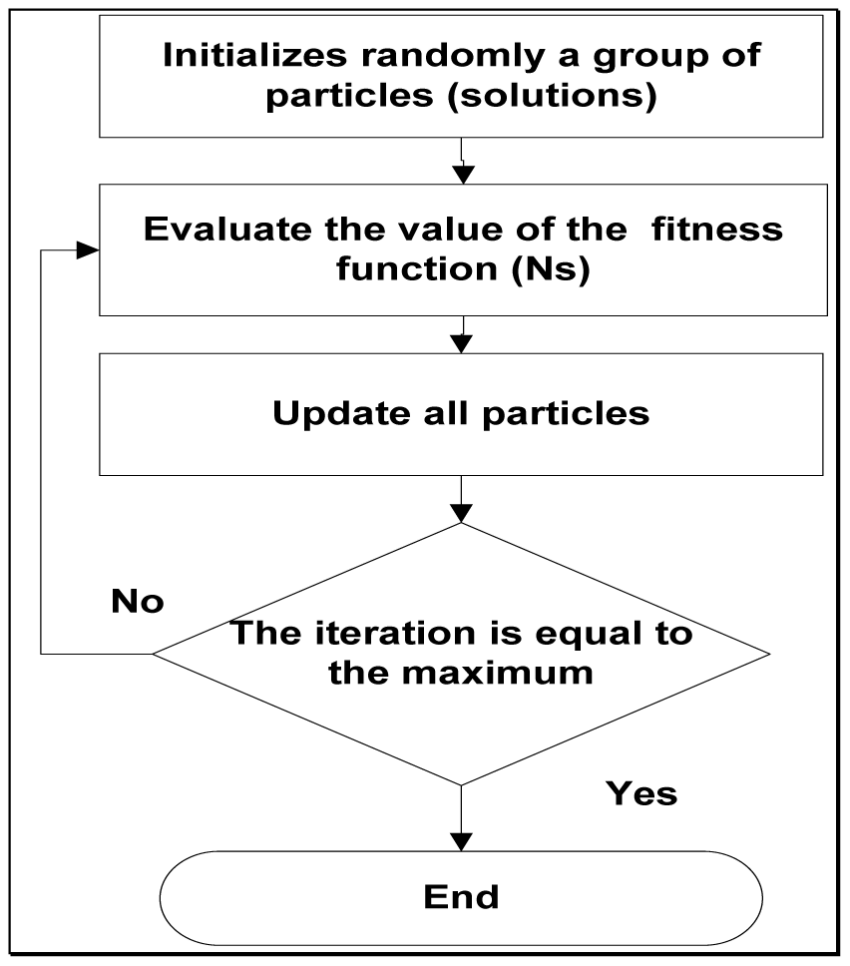
Flowchart of a PSO algorithm.

The presented Figure 1 shows the flowchart of PSO algorithm. Moreover, the general steps of the PSO technique [24], [40], [42] is given as follows:

- Initialize the particle s with random positions and velocities within the search space.
- Calculate the fitness value of each particle. In this study, the fitness value is calculated by dimensionless total entropy generation rate (***Ns***).
- Update the personal best position for each particle and the global best position for the population.
- Update the velocity and position of each particle.
- If termination criteria fulfils, stop else go back to step (iii).

Therefore, the particles (solutions) should move into better directions. In other words, they should intend to have been decreasing fitness as quickly as possible and exploit the useful information from some other particles besides the best particle [38], [42].

In this study, we use the PSO algorithm to minimize the noise of pin fin geometry. We set the range of 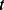
and 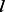 to be the interval (0, 0.88), while the range of 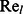
is the interval (1000, 3000). The total number of the iterations is set at 500 with a number of initial population as 60, and 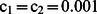 [38].

## Mathematical Analysis

**Figure 2:**
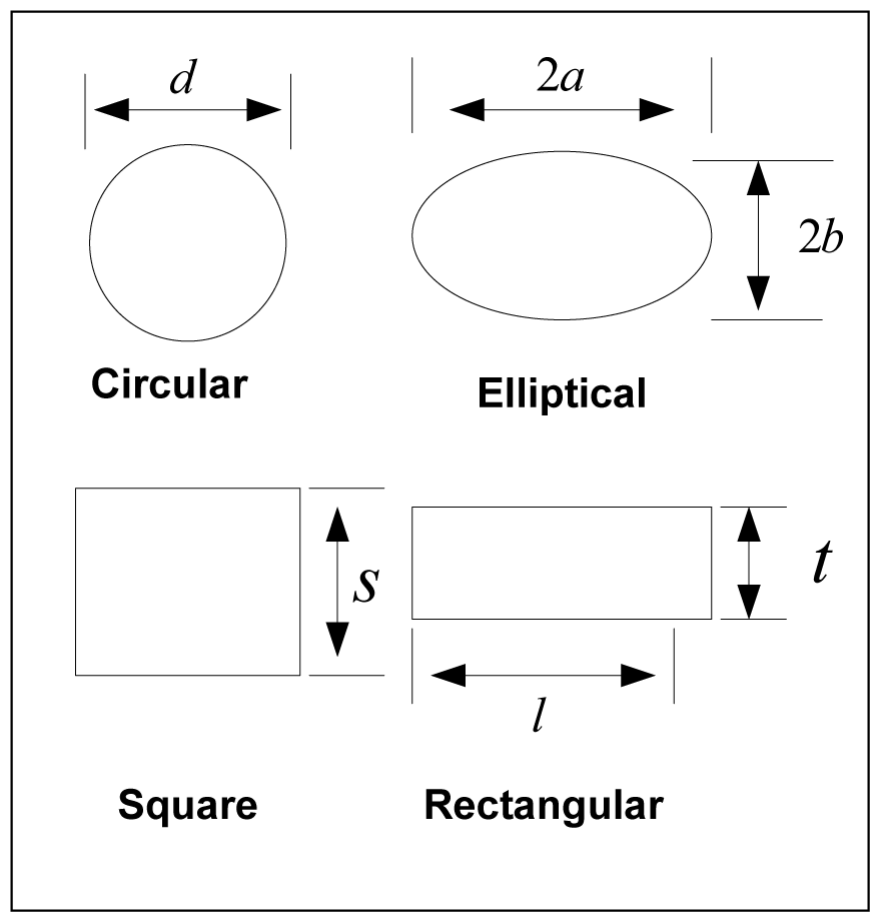
Cross-sections of different geometries.



Consider a pin fin of arbitrary cross section rectangular, circular, square, or elliptical as shown in Figure 2, which is extended from a base plate. It is assumed that there is no contact resistance between fin and the base plate. The fin material is assumed to be homogeneous and isotropic. The flow is assumed to be steady, laminar and two dimensional and the fluids are considered incompressible with constant properties. It is also assumed that there is no heat source within the fin itself and there is no radiation heat transfer from the fin. The approach velocity of the air is *U*_app_ and the ambient temperature of the air is assumed to be *T_a_*. The surface temperature of the pin wall is *T_w_*(>*T_a_*). Following Bejan [43] and Khan [10], the entropy generation rate can be written as

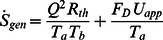
(3)where 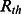
is the total thermal resistance and 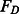
is the drag force and can be written as
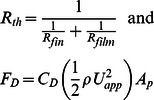
(4)


with
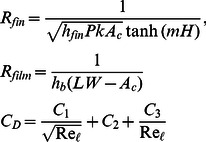
(5)


The fin parameter
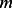
and the heat transfer coefficients for the fin and the base surface are given by
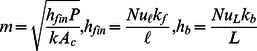
(6)


where Nusselt numbers for the base plate and the selected geometries were developed by khan [10] and are given by
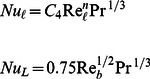
(7)


with
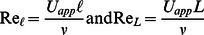
(8)


The constant 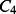 and the index 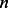 for the selected geometries are given in Table 1. In dimensionless form, entropy generation rate can be written as
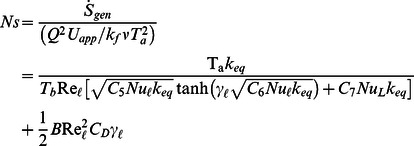
(9)


**Table 1:** Parameters for different geometries.

Geometry				
Parameters	Plate	Circular	Square	Elliptical
L	*l*	*d*	*s*	*2a*
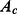	*tl*	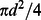	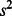	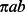
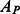	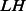	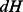	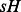	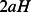
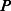	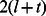	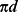	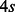	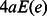
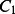	1.357	5.781	0	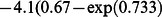
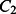	0	1.152	2	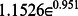
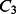	0	1.26	0	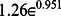
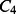	0.75	0.593	0.102	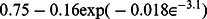
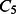	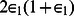	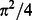	4	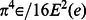
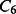	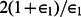	4	4	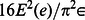
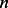	1/2	1/2	0.675	1/2

where
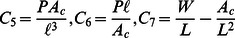
(10)


The values of these constants and the values of different parameters for the selected geometries are also presented in Table 1. The hypothetical cases to compare the overall performance of each fin geometry are illustrated in Table 2. The cross sections for different fin shapes are shown in Figure 2.

**Table 2:** Dimensions used to determine the performance of fin geometry.

Quantity	Dimension
Footprint (mm^2^)	50×50
Base plate thickness(mm)	2
Overall height of fin (mm)	12
Thickness of RPF (mm)	1
Approach velocity (m\s)	3
Thermal conductivity of solid (W/m·K)	237
Thermal conductivity of air (W/m·K)	0.026
Density of air (kg/m^3^)	1.1614
Specific heat of air (J/kg·K)	1007
Kinematic viscosity (m^2^/s)	1.58 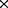 10^−5^
Prandtl number (Air)	0.71
Heat load (W)	10
Ambient temperature (K)	300
Base plate temperature (K)	350

## Results

**Figure 3:**
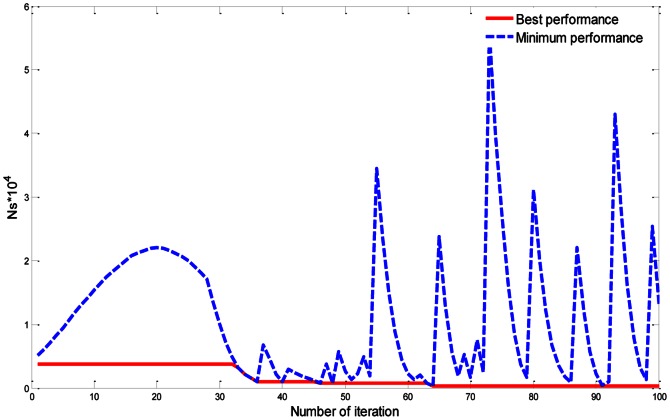
The global best performance and minimum performance of the algorithm per iteration in terms of *Ns*.

In the beginning, we have tried to make it clear about the technical way in the algorithm. In our analysis, the PSO algorithm was executed 50 times with 500 iterations each time. Then we reported the best results. In each iteration, there are many solutions, but the algorithm chooses the variables that give the best solution, then compare this with the best solution it has achieved so far (the best global value). In other words, the global best value is moved towards to a better solution. So, if all the solutions are moved towards better solutions, then, it can be stated that the algorithm can achieved convergence. Accordingly, the global best value will not change. For example, in Figure 3, we have determined the best global value and current minimum value of the dimensionless total entropy generation rate (***Ns***) per iteration. This is done by updating the search space of (P) in the interval (0, 30) and fixing 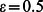 of rectangular plate fin (RPF). Thus, in each iteration the algorithm recorded the best global value. As mentioned, the solutions in PSO algorithm are called particles; these particles change their location by updating the distance depending on a term called velocity. Each particle is treated as a point within the search space, which must be specified. Each particle also carries a memory. In our analysis, the population size was set to be 60, which means that there are 60 solutions in each iteration. Furthermore, the best value of ***Ns*** was achieved when p =  28.679, as shown in Table 3.

**Table 3:** Updating p in RPF with 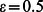.

p	*Ns*
28.679	1.1075e-6
22.882	1.393e-6
8.467	1.926e-6
4.0	4.085e-6
18.958	1.323e-6
27.436	1.129e-6

**Figure 4:**
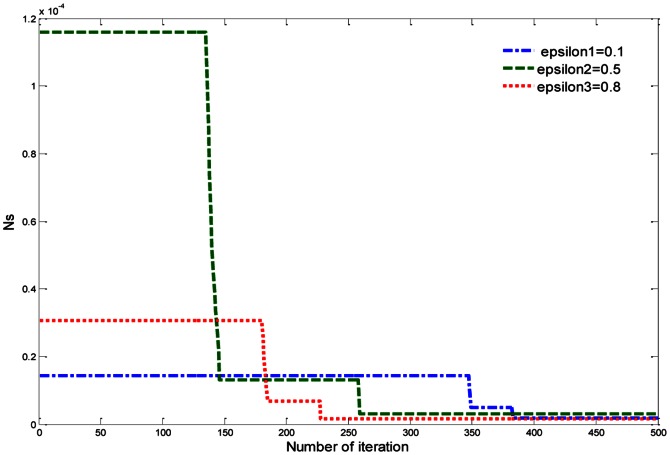
Effect of 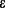 on dimensionless total entropy generation rate (*Ns*) of RPF.

In addition, the algorithm was executed 50 times with 500 iterations. By updating 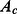
in PSO algorithm with different values of the aspect ratio (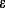) on the dimensionless total entropy generation rate (***Ns***) for rectangular plate fin (RPF) and elliptical pin fin (EPF), we found the following:

The minimum value of the dimensionless total entropy generation rate (***Ns***) for RPF is received when 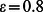, as shown in Table 4. In fact, the greater aspect ratio of RPF decreases the pressure drop and increases the heat transfer rate and as a result, the overall performance increases. It is clear that the overall performance of RPF increases with the aspect ratio, as shown in Figure 4.

**Table 4:** RPF with different values of 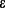.

ε	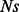	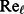
**0.1**	3.11E-06	3.00e+02
**0.5**	1.83E-06	3.23e+02
**0.8**	1.62E-06	2.02e+02

**Figure 5:**
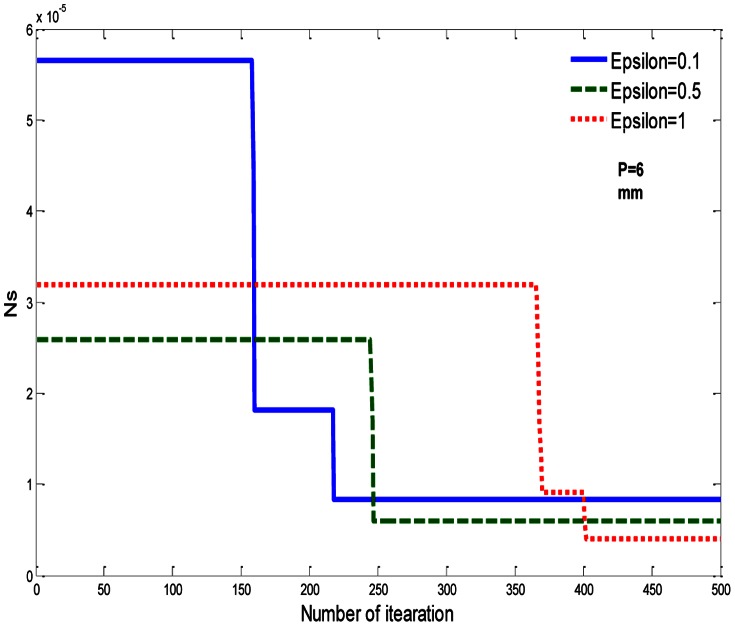
Effect of 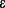 on dimensionless total entropy generation rate (*Ns*) of EPF.

On the other hand, the minimum value of the dimensionless total entropy generation rate (*Ns*) for EPF is obtained when 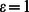, as shown in Table 5, while the effect of different values of 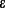 on ***Ns*** for EPF is illustrated in Figure 5.

**Table 5:** EPF with different values of 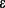.

ε	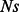	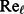
**0.1**	8.29E-06	1.67E+02
**0.5**	5.95E-06	1.87E+02
**1**	4.00E-06	4.48E+02

**Figure 6:**
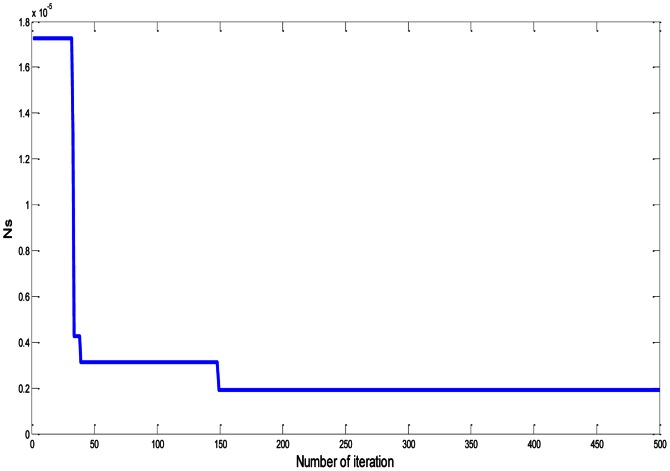
Dimensionless total entropy generation rate (*Ns*) of RPF with 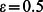 and P = 8.467.

**Figure 7:**
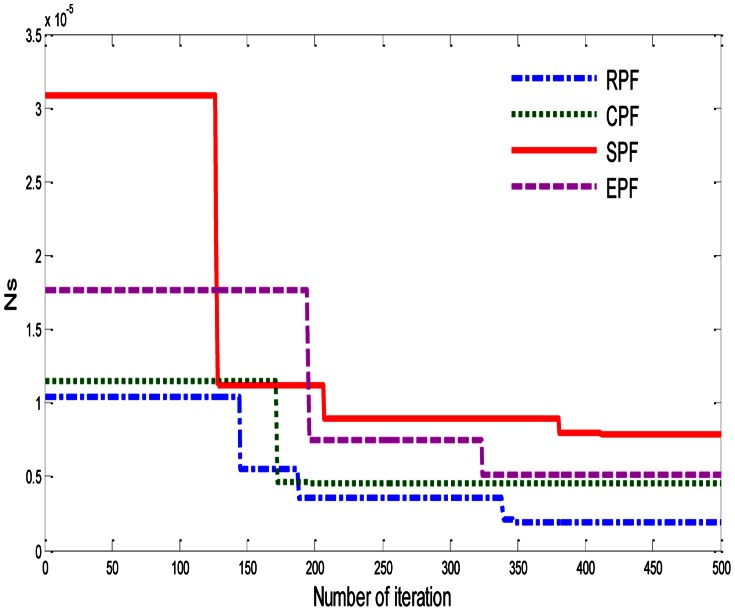
Effect of pin fin shape on dimensionless total entropy generation rate (*Ns*) for P = 6, 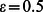, and 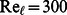.

**Figure 8:**
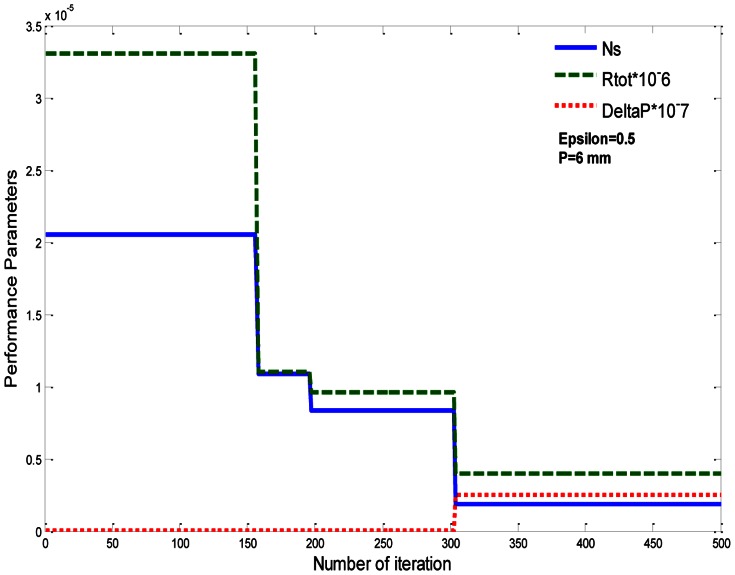
Variation of Ns, R_tot_, and ΔP for fixed values of P and ε.

In Figure 6, after 500 iterations, the value of (***N_S_***) is equal to 1.926e-6 and P = 8.467. The minimum values of ***N_S_*** for rectangular plate fin (RPF), circular pin fin (CPF), square pin fin (SPF), and elliptical pin fin (EPF), with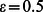, p = 6, and 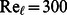
are illustrated in Table 6. We get these values also after 500 iterations, as shown in Figure 7. Figure 8 represents the relation between ***Ns***, R_TOT_, and ΔP, with p = 6 and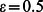. These results are better by comparing with the results in [44].

**Table 6:** The values of NS with 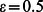.

Geometry	RPF	CPF	SPF	EPF
**Ns**	1.91e-06	4.57e-06	7.90e-06	5.11e-06

## Conclusions

Particle swarm optimization (PSO) has been employed successfully to investigate the overall performance of a pin fin with different cross sections. In order to combine the effects of thermal resistance and pressure drop, the idea of entropy generation minimization ( EGM) is employed. We have examined the effects of aspect ratio, Reynolds number, and perimeter of fin on the dimensionless total entropy generation rate. Optimal dimensionless entropy generation rate exists for each geometry. The square geometry is found to be have the worst choice from the point of view of total entropy generation rate. However, rectangular geometry is found to be the best. The results indicate that the preferred fin profile is very dependent on these parameters. It is found that PSO algorithm quickly converges to a good solution, and it is easy to obtain a local optimal value for each selected geometry.
